# Combining genetic and single-cell expression data reveals cell types and novel candidate genes for orofacial clefting

**DOI:** 10.1038/s41598-024-77724-9

**Published:** 2024-11-03

**Authors:** Anna Siewert, Simone Hoeland, Elisabeth Mangold, Kerstin U. Ludwig

**Affiliations:** Institute of Human Genetics, School of Medicine & University Hospital Bonn, University of Bonn, Bonn, Germany

**Keywords:** Cleft lip, Cleft palate, GWAS, scRNA-seq, hdWGCNA, Co-expression networks, Computational biology and bioinformatics, Developmental biology, Genetics

## Abstract

Non-syndromic cleft lip with/without cleft palate (nsCL/P) is one of the most common birth defects and has a multifactorial etiology. To date, over 45 loci harboring common risk variants have been identified. However, the effector genes at these loci, and the cell types that are affected by risk alleles, remain largely unknown. To address this, we combined genetic data from an nsCL/P genome-wide association study (GWAS) with single-cell RNA sequencing data obtained from the heads of unaffected human embryos. Using the recently developed single-cell disease relevance score (scDRS) approach, we identified two major cell types involved in nsCL/P development, namely the epithelium and the *HAND2*+ pharyngeal arches (PA). Combining scDRS with co-expression networks and differential gene expression analysis, we prioritized nsCL/P candidate genes, some of which were additionally supported by GWAS data (e.g., *CTNND1*,* PRTG*,* RPL35A*,* RAB11FIP1*,* KRT19*). Our results suggest that specific epithelial and PA sub-cell types are involved in nsCL/P development, and harbor a substantial fraction of the genetic risk for nsCL/P.

**Supplementary Information:**

The online version contains supplementary material available at 10.1038/s41598-024-77724-9.

## Introduction

Non-syndromic cleft lip with or without cleft palate (nsCL/P) is one of the most common birth defects, with a global prevalence of approximately 1 in 1,000 live births^1^. In addition to complex treatments such as surgery and speech therapy, affected patients are burdened by an increased risk of morbidity^2^. The etiology of nsCL/P involves both genetic and environmental factors^1^. To date, genome-wide association studies (GWAS) have identified more than 45 risk loci harboring common variants that are associated with increased nsCL/P risk^3^. At some loci, candidate genes have been pinpointed by evidence from syndromic forms of facial disorders, the presence of rare variants in affected individuals, or on the basis of results from animal models^4^. However, how the human risk variants affect the function of nsCL/P candidate genes and the cell types in which they are likely to act, remains largely unclear in most cases, although some initial work has been published^5–7^.

To address these questions, single-cell RNA-sequencing (scRNA-seq) is a promising approach. Instead of analyzing gene expression profiles in bulk from whole tissues, scRNA-seq enables the investigation of gene expression profiles in specific individual cells. When applied to biomaterials of relevance to specific diseases, this allows both the generation of high-resolution transcription maps of cell-types, and the identification of sub-cell types that might contribute to disease pathogenesis.

In the context of craniofacial development, most scRNA-seq studies to date have been performed on murine tissues and have identified cell types that would have been missed in earlier analyses of bulk data. For example, one study identified heterogeneity in gene expression of mesenchymal cells in the anterior palate^8^, while another found distinct cell populations at the fusion sites of the maxillary, medial-nasal, and lateral-nasal processes^9^ in mice. To study the role of these cell types in nsCL/P, we and others have utilized these murine single cell expression maps to examine gene expression patterns of candidate genes identified in genetic studies^10,11^. However, the suitability of murine data for the investigation of nsCL/P is limited. Reasons for this include: (i) differences in morphology and tissue interactions between mice and humans, in particular during the later stages of facial development^12^; and (ii) the fact that in humans, most genetic nsCL/P associations are located in non-coding (and often non-conserved) regions of the genome, indicating higher-order regulatory mechanisms^4^. Recently, scRNA-seq data from unaffected human embryos aged four to six weeks were made available^13^, which partly cover the crucial time period for nsCL/P development between the fourth and tenth week post-conception^14^.

The joint study of genetic and transcriptomic data has the potential to identify affected cell types and improve the understanding of disease mechanisms during facial development. To date, few computational approaches that combine genetic and single-cell transcriptomic data have been available. However, the recently developed single-cell disease relevance score (scDRS)^15^ now allows the identification of associations between candidate genes identified via GWAS and individual cells from scRNA-seq data. The aim of the present study was to identify human developmental cell types in which genetically-mediated nsCL/P risk is enriched, which is crucial in terms of unraveling the underlying molecular mechanisms of nsCL/P. For this purpose, the scDRS approach was used to combine scRNA-seq data of unaffected human embryos^13^ with candidate genes derived from our recent GWAS on nsCL/P^3^. The identified cell types were then used to determine potential interactions between candidate genes in co-expression networks using high-dimensional weighted correlation network analysis (hdWGCNA)^16^. We demonstrate how these approaches can facilitate the identification of molecular networks, effector cell-types, and novel candidate genes, thus advancing our understanding of the molecular basis of genetic nsCL/P risk.

## Methods

### Human embryonic scRNA-seq data

Human embryonic scRNA-seq data^13^ were downloaded from Gene Expression Omnibus (GSE157329, see data availability section). These data comprised scRNA-seq data from seven unaffected whole human embryos from Carnegie stage (CS) 12 (one embryo), CS 13–14 (three embryos), and CS 15–16 (three embryos), which had been broadly dissected into head, upper and lower trunk, limbs, and viscera. Based on meta-information provided by the authors, the data were reduced to dissection parts ‘head’ and ‘head-upperTrunk’. This led to the exclusion of two embryos without head data, i.e., one embryo respectively from CS 13–14 and CS 15–16. No additional filtering was performed. These data were then re-analyzed using Seurat v4.3.0^17^. Details on analysis parameters are provided in the Supplementary Information. Briefly, to remove potential batch effects, data from different samples were integrated using canonical correlation analysis, as implemented in Seurat. For this purpose, the data were split according to sample (*n* = 6, including one donor head that was split and analyzed as two samples), and processed as individual Seurat objects prior to integration.

**Fig. 1:**
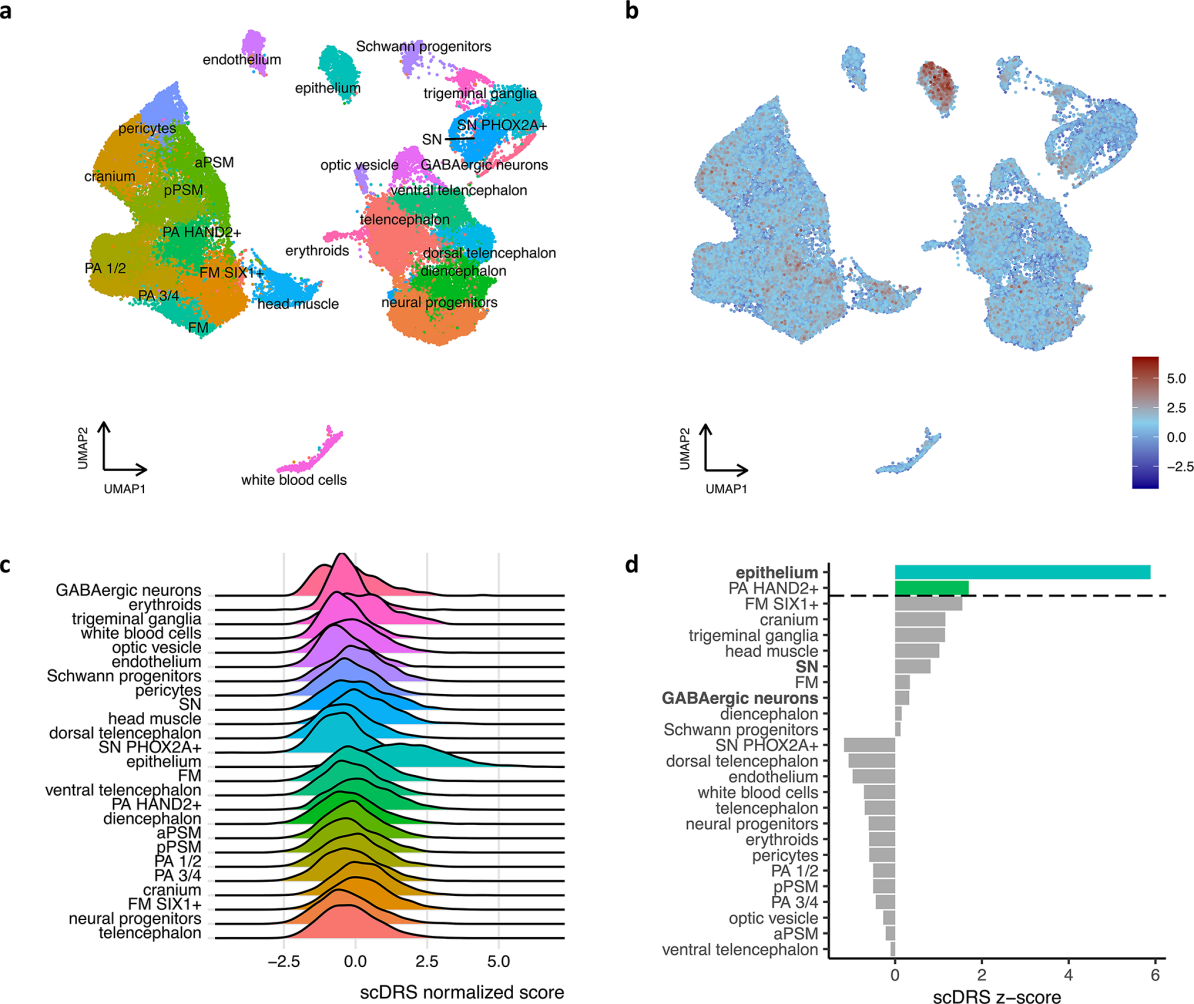
scDRS identifies significant association with nsCL/P candidate genes in epithelium and *HAND2*+ pharyngeal arches (**a**) UMAP plot of scRNA-seq data from the heads of five unaffected human embryos from Carnegie stages 12–16. (**b**) UMAP plot from **a** colored according to the normalized scDRS for nsCL/P association at the single-cell level in the unweighted setting. (**c**) Ridgeplot of normalized scDRS according to cell type in the unweighted setting. (**d**) scDRS disease association at the cell type level in the unweighted setting. Cell types above the dashed line showed significant association with the nsCL/P gene set. Bold cell type labels indicate significant within-cell type heterogeneity in terms of disease association. Anterior presomitic mesoderm (aPSM), frontonasal mesenchyme (FM), log_2_ fold change (log_2_FC), pharyngeal arches (PA), posterior presomitic mesoderm (pPSM), sympathetic neurons (SN), single-cell disease relevance score (scDRS).

The data were normalized and 2,000 highly variable genes were identified before the data were scaled. For the integration of individual Seurat objects, integration anchors between objects were identified and then used to integrate the individual data sets into one data set. The resulting data set was scaled and cell cycle regression was performed as implemented in Seurat. Principal component analysis was performed using the variable features of the data. Clustering was performed by first identifying the shared nearest neighbors of cells and then clustering the cells using the original Louvain algorithm. The resulting data set contained 50,059 cells, which clustered into 25 cell clusters (between 276 and 4,993 cells per cluster, Fig. 1A). The clustering showed no influences attributable to sample batch effects (Fig. S1A). Cluster marker genes were determined (Table S1) and used for cell type annotation of the clusters, as based on the cell type marker genes from the original publication (Supplementary Table S1B from Xu et al. 2023). Identification of differentially expressed genes (DEGs) was performed for epithelial sub-clusters only (Table S2).

### Identification of nsCL/P candidate cell types using scDRS

#### Preparation of the scRNA-seq data

To render the scRNA-seq data applicable for scDRS, the scaled data was removed and the Seurat object (containing only count and normalized data) was converted into an .h5ad file. Specific parameters are provided in the Supplementary Information.

#### Definition of nsCL/P gene set

For the preparation of the genetically-informed gene set, the candidate genes located in topologically associating domains (TADs) of nsCL/P GWAS risk loci were retrieved from Table S9 from Welzenbach et al. 2021^3^. Of the 404 genes located in these TADs, 51 were not detected in the scRNA-seq data. The remaining 353 genes are referred to as ‘TAD genes’. Of these, 87 genes had reached significance in the gene-based test in the original publication, thus providing further genetic support for these genes beyond single-variation association statistics at the risk loci. These genes were used for scDRS analysis, and are referred to as ‘nsCL/P gene set’. For all of these genes, z-scores were retrieved from the MAGMA output file from Welzenbach et al. 2021^3^, and were used as weights in the “weighted setting”.

#### Application of the scDRS method

Following download (GitHub) and installation of the scDRS package^15^, scDRSs for all single cells in the scRNA-seq data (*n* = 50,059) were calculated. For the nsCL/P gene set, this was performed in both settings, i.e., “unweighted” and “weighted”. Downstream analyses were conducted on the scores generated from each gene set. Specific analysis parameters are provided in the Supplementary Information.

#### Additional quality assessment

To identify potential artifacts, the entire scRNA-seq data set was used. Here, Pearson correlation between the scDRS of each cell and the total number of detected molecules of each cell was calculated.

### Co-expression network analysis using hdWGCNA

To generate co-expression networks, the hdWGCNA package version 0.3.1 was used (see section Data availability). First, metacells were created from the single cell matrix, which were then normalized. Next, for both the epithelium and the *HAND2*+ PA, an expression matrix of the respective metacells was constructed. The soft power thresholds were determined, the co-expression networks were constructed and the matrices were scaled. Then, module eigengenes and eigengene-based connectivity were determined. Specific analysis parameters are provided in the Supplementary Information. For each gene module, the percentage of TAD genes was calculated, and the three top gene modules, i.e., those with the highest percentage of TAD genes, were selected for further analysis. The Circos plots for the co-expression gene modules were created using the R package circlize^18^.

### Gene ontology enrichment analysis

A gene ontology (GO) analysis was performed using the clusterProfiler^19^ and org.Hs.eg.db^20^ R packages to identify biological process and molecular function GO terms. For E-9 and PA-14, redundant GO terms were removed (*simplify* function). Specific analysis parameters are provided in the Supplementary Information.

### Identification of new candidate genes using hdWGCNA gene modules and GWAS data

For genes that were identified in the hdWGCNA gene modules, p-values from the gene-based test in our recent GWAS were retrieved from Table S6 from Welzenbach et al. 2021^3^. FDR correction was performed using the Benjamini Hochberg method. For those genes that remained significant (adjusted p-value < 0.05) and were located outside of nsCL/P GWAS TADs, LocusZoom^21^ was used to create regional association plots from the GWAS summary statistics^3^. To assess whether the co-expression gene modules were enriched for genes with a genetic association to nsCL/P, a gene set analysis was performed using MAGMA v1.10^22^ (see section Data availability). The gene sets were created from the six selected co-expression modules (E-9, E-10, E11, PA-12, PA-14, PA-15). The analysis was performed using these gene sets and the MAGMA genes.raw output file from Welzenbach et al. 2021^3^.

## Results

### Expression patterns of GWAS candidate genes implicate head epithelium and *HAND2*+ pharyngeal arches in genetically-mediated nsCL/P

Based on scRNA-seq data from the heads of unaffected human embryos (Fig. 1A) and the nsCL/P gene set informed by GWAS results (see Methods), developmental cell types that might underlie nsCL/P etiology were identified using scDRS.

First, scDRSs were calculated for each single cell in the scRNA-seq data (*n* = 50,059) in two settings, i.e., unweighted and weighted (by MAGMA z-scores, see Methods). At the single-cell level and across the two settings, an accumulation of cells with high scDRS was observed in the epithelium (Fig. 1B,C, Fig. S1B). When combining the scDRSs of individual cells over cell-clusters, this accumulation in the epithelium was found to be statistically significant in both the unweighted (*p* = 0.002, Fig. 1D, Table S3) and the weighted setting (*p* = 0.01, Fig. S1C, Table S4). In addition, the cell type *HAND2*+ PA reached statistical significance in the unweighted analysis (*p* = 0.04; Fig. 1D, Table S3). To identify potential subpopulations of disease-associated cells, all cell types were tested for evidence of within-cell type heterogeneity. In the unweighted setting, significant within-cell type heterogeneity was observed in the epithelium, as well as in sympathetic and GABAergic neurons (Fig. 1C). In the weighted setting, significant within-cell type heterogeneity was observed in the dorsal telencephalon, the sympathetic neurons, the endothelium, and the GABAergic neurons (Fig. S1C).To ensure that the scDRS for each cell and thereby the heterogeneity in association was not caused by technical differences in transcript detection, the correlation between the scDRS of each cell and the total number of molecules detected was tested. No strong support for such a technical bias was found (Pearson correlation coefficient: 0.006).

**Fig. 2:**
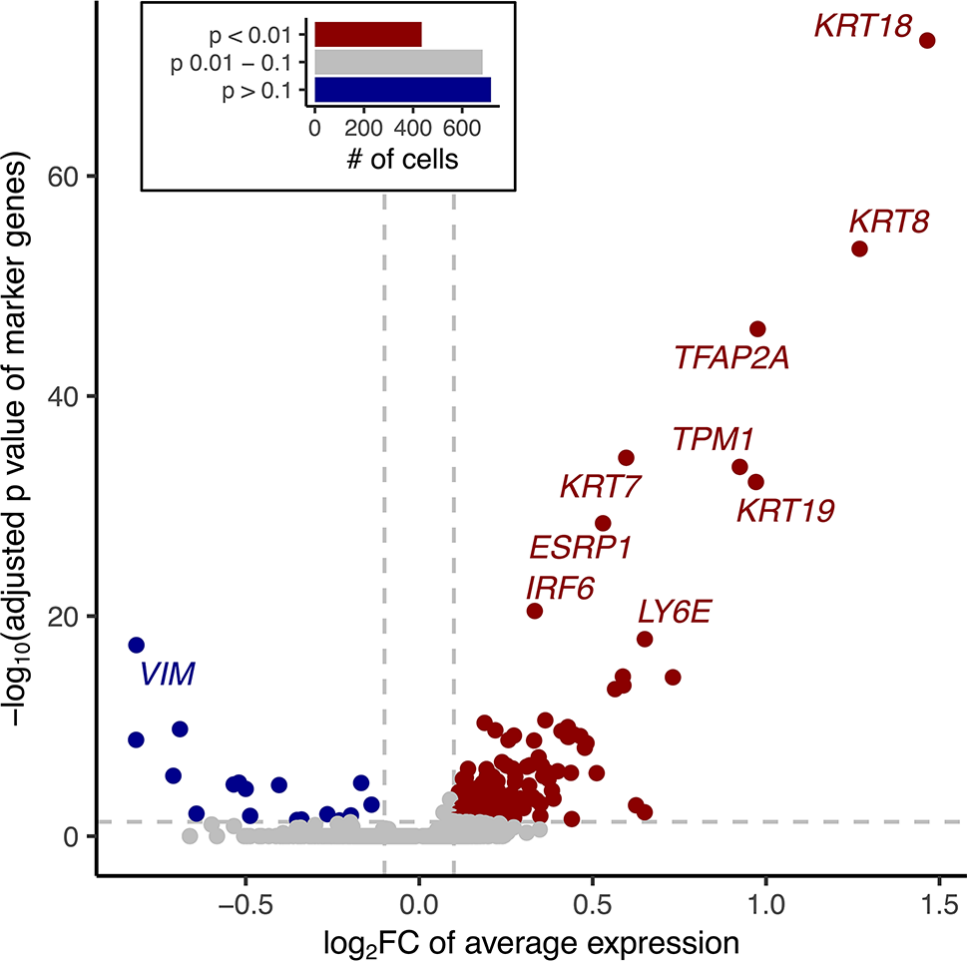
Marker genes of associated epithelial cells contain known nsCL/P candidate genes. Volcano plot showing differentially expressed genes between nsCL/P associated (scDRS p-value < 0.01) and non-associated (scDRS p-value > 0.1) epithelial cells. Numbers within each group are depicted in the integrated bar plot. Genes with adjusted p-values < 0.05 (dashed horizontal line) and log_2_FC > 0.1 were considered marker genes for nsCL/P associated cells (red). Genes with adjusted p-values < 0.05 and log_2_FC < -0.1 were considered marker genes for non-associated cells (blue). Top 10 genes with the lowest p-values are labeled. Non-syndromic cleft lip with/without cleft palate (nsCL/P), log_2_ fold change (log_2_ FC), single-cell disease relevance score (scDRS).

Given the converging evidence for a role in nsCL/P, and potential heterogeneity within the epithelial cell cluster, this cell cluster was then subdivided into two subclusters, as based on the scDRS p-value of each cell. This resulted in the identification of an associated subcluster (435 cells, *p* ≤ 0.01) and a non-associated subcluster (717 cells, *p* ≥ 0.1), from a total of 1,835 cells. DEGs between these subclusters were then identified. A total of 139 DEGs showed higher expression in the subcluster of disease-associated cells (fold change > 1) compared to the subcluster containing non-associated cells (fold change < -1; Fig. 2; Table S2). Of these, 31 genes were among the 353 ‘TAD genes’, and 25 of these 31 genes were among the 87 genes of the ‘nsCL/P gene set’ of previously suggested effector genes (e.g., *KRT8*,* KRT18*,* TFAP2A*,* TPM1*,* ESRP1*, and IRF6; Fig. 2; Table S2). Based on these results, we prioritized the remaining six TAD genes as nsCL/P candidate genes (Table 1, ‘DEG’ approach). Notably, for some of them, evidence of an involvement in orofacial clefting phenotypes has already been presented, though not from GWAS data^23–25^.

**Table 1:** Summary of potential new candidate genes for nsCL/P based on different analysis approaches.

Analysis approach	Prioritized nsCL/P candidate genes
scDRS TAD gene	***RPL35A***, ***C10orf82***, *MSX1*, ***GADD45B***, *ARHGAP29*, ***TLE2***, ***NSD3***, ***TIMM13***, ***RAB11FIP1***, ***NBL1***
scDRS gene	***KRT19***, ***CLDN6***, ***EPCAM***, ***CLDN4***, ***CLDN7***, ***RAB25***, ***RPS14***, ***RPL41***, ***RPS18***, ***AP1M2***
hdWGCNA + GWAS	*CTNND1*,* PRTG*, ***BFAR***, ***HYAL2***
DEGs	*ARHGAP29*,* MYC*, ***GADD45B***,*** RAB11FIP1***,*** PLKHF2***,*** NSG1***

The top 10 genes per analysis approach are shown, the remaining genes for scDRS TAD gene and scDRS gene are listed in table S5. Genes that have not been previously reported with nsCL/P are shown in bold.

Analysis approaches are defined in the main text.

Abbreviations: Differentially expressed genes (DEGs), genome-wide association study (GWAS), high-dimensional weighted correlation network analysis (hdWGCNA), non-syndromic cleft lip with or without cleft palate (nsCL/P), single-cell disease relevance score (scDRS), topologically associating domain (TAD).

### scDRS-informed prioritization of candidate genes at GWAS loci

In addition to confirming known nsCL/P risk genes, scDRS within associated cell types can also be used to identify potential novel candidate genes. For this purpose, we used the gene-level downstream application of scDRS, which correlates the scDRS with the expression of genes that are not part of the tested gene set. Specifically, we aimed to identify genes at GWAS loci that were not prioritized as candidate genes due to the presence of another promising gene, or due to the lack of a significant gene-based P-value in the genetic data^3^. In this analysis, we considered genes with a correlation coefficient of > 0.01. In the unweighted setting, a positive correlation was observed between gene expression and scDRS for 33 genes (Table 1 ‘scDRS TAD gene’ approach, Table S5), with the highest absolute value being observed for *RPL35A* (Pearson correlation coefficient: 0.15). This gene is located at the 3q29 locus, which harbors the previously proposed candidate genes *DLG1* and *MELTF.* The TAD genes *GADD45B* (19p13.3), *ARHGAP29* (1p22), and *MSX1* (4p16.2) showed a positive, albeit less pronounced correlation (Pearson correlation coefficients between 0.048 and 0.055). Our findings provide additional support for prioritizing these as effector genes at their respective loci. Similar results were found in the weighted setting (Table S6).

### Co-expression network analysis of epithelium and *HAND2*+ pharyngeal arches

To identify genes with potential interactions in the previously identified nsCL/P-associated cell types (epithelium and *HAND2*+ PA), co-expression networks were generated. For each cell type, hdWGCNA identified 18 groups of interconnected, positively correlated genes (so-called ‘gene modules’). Of these, three per cell type were selected for further analysis (see Methods, Tables S7 & S8).

**Fig. 3:**
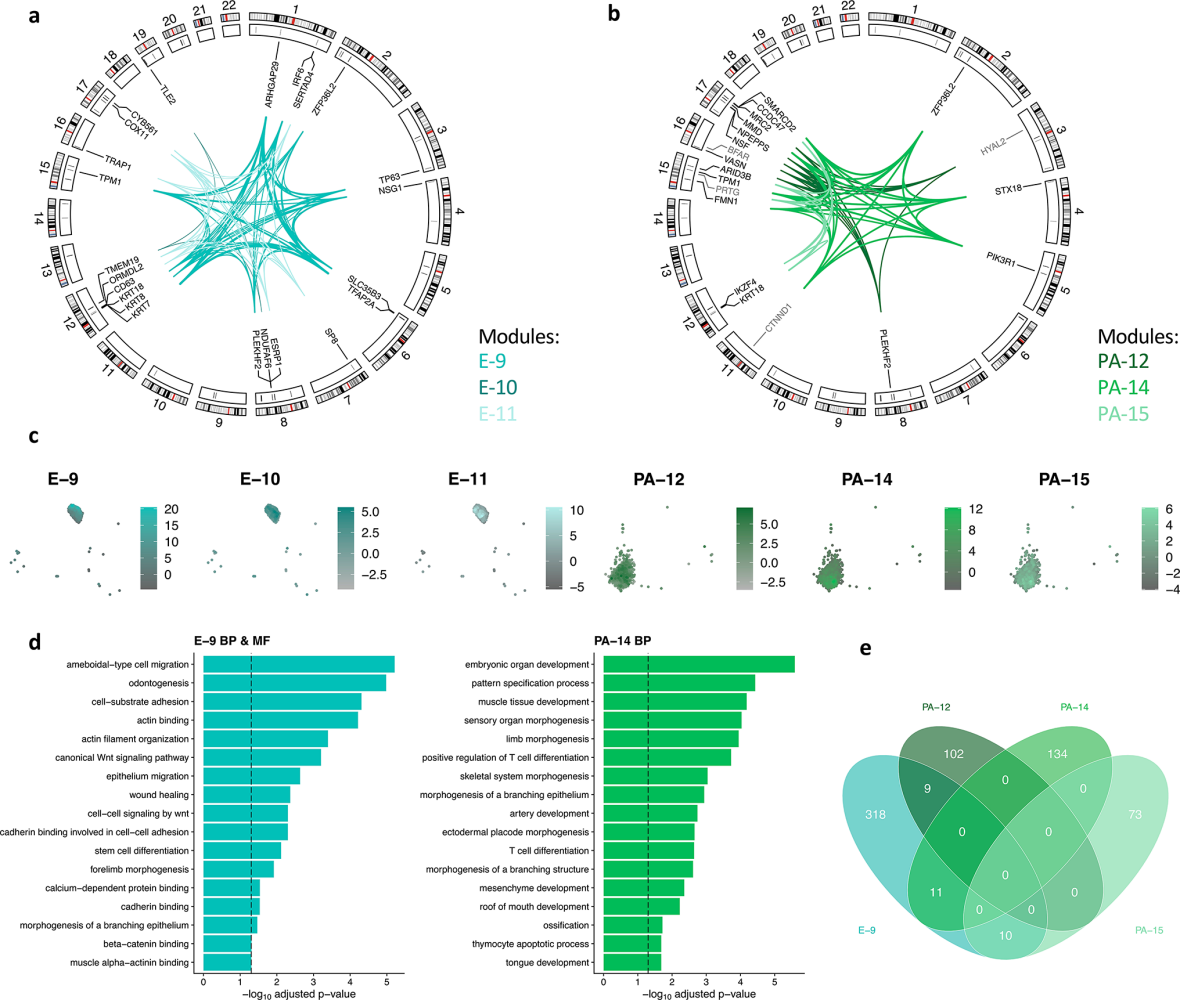
Co-expression gene modules of epithelium and *HAND2*+ PA. (**a**) Circos plot of nsCL/P genes in epithelial co-expression gene modules. The outer track shows the chromosomal cytoband, the inner track shows the positions of TADs described in Welzenbach et al. 2021^3^. The colors of the connecting lines correspond to the respective gene module. The strength of the connecting lines reflects the pairwise correlation coefficient between two genes multiplied by a factor of 30 for illustration purposes. (**b**) Circos plot of nsCL/P genes (black) and potential novel candidate genes (gray) in *HAND2*+ PA co-expression gene modules. Panel layout as described in **a**. (**c**) UMAP plots of epithelium (E-9, E-10, E-11) and *HAND2*+ PA (PA-12, PA-14, PA-15) colored according to the module eigengene values for each co-expression gene module. (**d**) Bar plots of selected GO terms for biological process (E-9 & PA-14) and molecular function (E-9) for the epithelial co-expression gene module E-9 and the *HAND2*+ PA co-expression gene module PA-14. The vertical dashed line is set at p-value -log_10_ of 0.05. (**e**) Venn diagram of gene overlap between epithelial gene module E-9 and *HAND2*+ PA gene modules PA-12, PA-14, and PA-15. Epithelium (E), gene ontology (GO), non-syndromic cleft lip with/without cleft palate (nsCL/P), pharyngeal arches (PA), topologically associating domains (TADs).

Of the epithelial gene modules, the following were selected: E-9 (348 genes / including 12 nsCL/P TAD genes); E-10 (73/3); and E-11 (201/8) (Fig. 3A, Table S7). The eigengene values within the epithelial cluster were then plotted in their UMAP space of scRNA-seq data (Fig. 3C). While for E-9 and E-11, these appeared to be restricted to the upper part and lower part of the UMAP plot respectively, the highest values for E-10 did not appear specific. For E-9, the hub genes (i.e., genes with the largest number of connections within the module’s network) included the nsCL/P TAD genes *TFAP2A*, *TPM1*, and *ARHGAP29*, thus providing further support for the hypothesis that they play a causal role in nsCL/P at their respective loci (Table S9)^24,26^. Enrichment analyses for E-9 using GO terms identified odontogenesis, wound healing, actin filament organization, the canonical Wnt signaling pathway, and Cadherin binding (Fig. 3D, Table S10). In contrast to E-9, the hub genes of E-10 and E-11 contained no nsCL/P TAD genes, and the GO term analysis results were non-specific (pituitary gland development and central nervous system neuron differentiation for E-10; regulation of neuron differentiation, ribosome binding, and unfolded protein binding for E-11; Table S10). Together, this suggests that the most relevant epithelial gene module in terms of nsCL/P risk may be E-9.

For *HAND2*+ PA, the selected gene modules were: PA-12 (111 genes / including 5 nsCL/P TAD genes), PA-14 (145/7), and PA-15 (83/4; Fig. 3B, Table S8). The highest module eigengene values for PA-12 and PA-15 were distributed evenly over the cluster, while the highest values for PA-14 were more concentrated at the bottom of the UMAP space (Fig. 3C). For PA-12, the identified hub genes included *MRC2*, which is located at the nsCL/P risk locus 17q23.2 but showed no significant gene-based association in Welzenbach et al. 2021^3^. Notably, while a set of seven genes is located at this locus, none has garnered sufficient research evidence to date to be considered the effector gene. Therefore, our results now prioritize *MRC2* as a candidate gene for functional studies. The hub genes of PA-14 contained the nsCL/P candidate genes *TPM1* and *ZFP36L2*, while those in PA-15 included the nsCL/P candidate gene *KRT18* (Table S11). The GO term analysis generated no significantly enriched terms for PA-12 or PA-15 (Table S10). However, for PA-14, the GO terms included T-cell differentiation and the development of the roof of the mouth, tongue, muscle tissue, arteries, and mesenchyme (Fig. 3D, Table S10).

When comparing the three epithelial gene modules and the three *HAND2*+ PA gene modules, a limited overlap of between 1 and 11 genes was observed (Fig. 3E, Fig. S2A/B, Table S12 e.g. *ZFP36L2* and *TPM1*). This suggests that most genes located at GWAS loci act or interact in only one of the two cell types.

### MAGMA gene set analysis identifies association between epithelial co-expression gene module and nsCL/P

To examine the joint association of genes within hdWGCNA-identified co-expression gene modules and nsCL/P, a MAGMA gene set analysis was performed using the six selected gene modules as individual gene sets (see above) and the nsCL/P GWAS summary statistics from Welzenbach et al. 2021^3^. This analysis revealed a significant association with nsCL/P for gene module E-9 (*p* = 5.98 × 10^− 5^), and provides further evidence that this gene module is enriched with genes that are associated with nsCL/P (Table S13).

### Identification of novel nsCL/P candidate genes

To identify potential novel nsCL/P candidate genes, an analysis was performed of genes that are located outside the GWAS-TADs and which represent plausible novel candidate genes given similarities in scDRS and co-expression networks. First, genes whose expression patterns are positively correlated (correlation coefficient > 0.01) with the scDRS of individual cells (see above), but which are located outside of any known GWAS locus, were identified. Here, *KRT19*, *EPCAM*, and the Claudin family members *CLDN6*, *CLDN4*, and *CLDN7* were moderately correlated with the scDRS in the unweighted setting (Pearson correlation coefficients 0.17 to 0.29; Table 1 ‘scDRS gene’ approach, Table S5). None of these genes showed significance in the MAGMA gene-based test in Welzenbach et al. 2021^3^. However, *KRT19* and *EPCAM* were among the epithelial co-expression modules (E-9 and E-11, respectively). Similar results were obtained for the weighted setting (Table S6).

Second, genes that were listed among the selected hdWGCNA gene modules (E-9, E-10, E-11, PA-12, PA-14, and PA-15), and which were significant in the gene-based test, were examined. No new candidate genes were identified in any of the three epithelial gene modules. However, evidence was generated to suggest that *HYAL2* and *BFAR* (both in PA-12), *CTNND1* (PA-14), and *PRTG* (PA-15) represent novel nsCL/P candidate genes from the *HAND2*+ PA gene modules (Table 1 “hdWGCNA + GWAS’ approach, Table S8). An examination of the association structure around these genes in the GWAS summary statistics yielded nominally significant genetic support for the loci harboring *CTNND1* and *PRTG* (Fig S2 C/D). This suggests that these loci might reach conservative thresholds for genetic associations of common variation in future studies involving increased power. Interestingly, previous studies already linked rare variants in *CTNND1*^27^ and low-frequency coding variants in *PRTG*^28^ to nsCL/P.

## Discussion

In recent years, multiple genetic studies on nsCL/P have identified genomic risk loci, and suggested local candidate genes in the associated regions^3,25,29–43^. However, since most of the associated regions map to non-coding parts of the genome and can thus be presumed to have context-specific effects, biological interpretation of these discoveries requires the identification of the affected cell types. This knowledge would in turn inform the context in which functional studies should be performed, which is essential for understanding the molecular mechanisms of nsCL/P development. For some established candidate genes, expression patterns have already been reported, e.g., *IRF6* expression in neural crest and epithelial cells^6,44–48^, and *TFAP2A* expression in facial mesenchyme, nervous system, epithelial, and neural crest cells^49–51^. In addition, in a previous study involving single-cell transcriptome analyses in mice^10^, our group showed that the murine homologs of certain nsCL/P candidate genes are expressed predominantly in either epithelial cell types (e.g., candidate genes *IRF6*, *TFAP2A*, *ESRP1*) or mesenchymal-like cell types (e.g., candidate genes *ALX1*, *ALX3*, *GREM1)*. The present study complemented previous research by performing a systematic examination of the joint gene expression of nsCL/P candidate genes from GWAS, with the aim of detecting the human developmental cell types that mediate genetic nsCL/P risk.

Based on expression data from unaffected human embryonic heads, our scDRS analysis implicated the epithelium and *HAND2*+ PA as primary cell types with an involvement in genetic nsCL/P risk. This confirms, and further refines, observations from our previous study in mice, which showed that individual nsCL/P genes were expressed in epithelial and mesenchymal cell types^10^. Epithelial cells are involved in manifold processes during lip and palate formation. These processes include: (i) epithelial seam formation, which is required for the fusion processes of the upper lip and the palate, as well as those between the medial-nasal, the lateral nasal, and the maxillary prominences^52–55^; (ii) epithelial-to-mesenchymal transitions, which allow for movement of cells as facial prominences grow, as well as the removal of epithelial seams^56–58^; (iii) the formation of the periderm, which covers the developing epithelium^59^; and (iv) cell adhesion and migration^60,61^. We speculate that the heterogeneity we observed in the overall epithelial cell cluster might recapitulate different expression patterns associated with these different functions. Indeed, one of the top markers of the associated epithelial cells is *IRF6*, which is a particularly relevant gene in periderm formation, and has been shown to be crucial in the development of the palate^62,63^. However, we note that the data include the whole head and, therefore, the possibility remains that the within-cell type heterogeneity might also be caused by epithelial cells originating from other regions of the head, rather than the facial processes. The second major cell-type we identified were *HAND2*+ PA, one of three clusters that were annotated as PA, which give rise to the bones and connective tissue of the head^64^. The transcription factor *HAND2*, which characterized the specific PA cluster associated with nsCL/P, was previously found to be expressed in the neural-crest derived mesenchyme of the PA of mice^65^ and involved with patterning in the PA of zebrafish^66^. Together, the findings of specific cell types within PA and epithelium suggest that there may be specific sub-cell types that are involved in nsCL/P, which should be assessed with spatial and functional data in future studies.

Having identified relevant cell-clusters, the respective expression data can be explored using co-expression network analysis in order to identify genes that are potentially subject to the same gene regulation or which interact on a molecular level. This can help to prioritize effector genes at established genomic risk loci or identify new candidate genes. Importantly, our co-expression modules identified known interactions, such as *IRF6* and *TFAP2A*, which have been shown to act jointly in a genetic pathway^5,6^, as well as *IRF6* and *TP63*, the latter of which has been reported to activate *IRF6* expression^67^. This suggests that some of the newly identified genes within the same gene modules are promising genes for further functional studies, for example, *TPM1* and *ZFP36L2*, which occurred in the same gene module in the epithelial cells and in the *HAND2*+ PA. Interestingly, recent studies found that *ZFP36L2* was significantly associated with nsCL/P and one of its subtypes, i.e., non-syndromic cleft lip only, in GWAS data from a Chinese Han population^68,69^. We also found evidence for a role of *RPL35A*, which is located in a larger deletion region in patients with craniofacial abnormalities^70^. Our data provide further support for the suspected genes *GADD45B*, *ARHGAP29*, and *MSX1*, though these had not been prioritized in Welzenbach et al. 2021^3^. The present analyses also identified new candidate genes located outside of GWAS loci, such as *CTNND1* and *PRTG*, which were implicated through their expression patterns in the *HAND2*+ PA gene modules. For both genes, genetic support is provided by our in-house GWAS data, but also through rare variants identified by exome sequencing of multiplex families for *CTNND1*^27^ and low-frequency coding variants in *PRTG*^28^.

To obtain information on the biological relevance of the gene modules, we performed GO term analyses. The results reflect the before discussed processes the epithelium and PA are involved in, e.g. epithelial morphogenesis, mesenchyme development, and migration. Additionally, they support functional hypotheses such as cadherin-binding via *CTNND1*, involvement of several members of the Wnt-family, and muscle tissue development, all of which have been previously implicated in nsCL/P^27,71–78^. The association of one gene module with T-cell differentiation could provide an exciting link to immunological factors, which requires further examination. The differences in biological functions between the gene modules of epithelium and PA together with a very small gene overlap in genes between the gene modules, suggest that the genetic risk for nsCL/P is split on different biological, and maybe complementary, functions across those two cell types.

In summary, we combined human scRNA-seq data with genetic information on nsCL/P risk and identified nsCL/P-associated cell types and potential sub-cell types, which might harbor a considerable part of the genetic risk. Co-expression networks in these cell types allowed us to identify established and potential new gene-gene interactions. We also demonstrated how to identify new candidate genes based on these networks by revisiting the initial GWAS data.

## Electronic supplementary material

Below is the link to the electronic supplementary material.

Supplementary Material 1

Supplementary Material 2

## Data Availability

The original scRNA-seq data from Xu et al. are available via Gene Expression Omnibus accession number GSE157329 or via https://www.ncbi.nlm.nih.gov/geo/query/acc.cgi?acc=GSE157329. Our re-analyzed scRNA-seq data have been deposited at Zenodo in Seurat object format (DOI: 10.5281/zenodo.12742819). hdWGCNA documentation: https://smorabit.github.io/hdWGCNA/index.html. MAGMA: https://cncr.nl/research/magma/.
