# Supplementary material for: Combining genetic and single-cell expression data reveals cell types and novel candidate genes for orofacial clefting
Source: Sci Rep. 2024 Nov 3;14:26492. doi: 10.1038/s41598-024-77724-9 (PMC11532359; doi:10.1038/s41598-024-77724-9)
Supplement: Supplementary file 1 — Supplementary Material 1 [file 41598_2024_77724_MOESM1_ESM.pdf]

Combining genetic and single-cell expression data reveals cell types and novel candidate genes for orofacial clefting

Anna Siewert[1], Simone Hoeland[1], Elisabeth Mangold[1], Kerstin U. Ludwig[1]

[1]Institute of Human Genetics, University of Bonn, School of Medicine & University Hospital Bonn, Bonn, Germany

Supplementary Methods

This section describes the parameters used for the computational analyses.

1) Human embryonic scRNA-seq data analysis using Seurat

Function	Parameters
NormalizeData	normalization.method = “LogNormalize”, scale.factor = 10000
FindVariableFeatures	selection.method = “vst”, nfeatures = 2000
FindIntegrationAnchors	anchor.features = 2000, dims = 1:30
IntegrateData	dims = 30
FindNeighbors	dims = 25
FindClusters	resolution = 1
FindAllMarkers	only.pos = TRUE, min.pct = 0.25, logfc.threshold = 0.25
FindMarkers	only.pos = FALSE, min.pct = 0.01, logfc.threshold = 0.01

2) Preparation of Seurat object data for scDRS using SeuratDisk

Function	Parameters
DietSeurat	counts = TRUE, data = TRUE, scale.data = FALSE
Convert	dest = “h5ad”

3) Identification of nsCL/P candidate cell types using scDRS

Function	Parameters
compute score	--h5ad-species human, --gs-species human, --flag-filter-data True, --flag-raw-count False, --n-ctrl 1000, --flag-return-ctrl-raw-score False, --flag-return-ctrl-norm-score True
perform downstream	--gene-analysis, group-analysis seurat_clusters, --flag-filter-data True, flag-raw-count False

4) Co-expression network analysis using hdWGCNA

Function	Parameters
SetUpForWGCNA	gene_select = "fraction", fraction = 0.05, group.by = "seurat_clusters"
MetaCellsByGroups	group.by = "seurat_clusters", reduction = "pca", k = 50, max_shared = 20, ident.group = "seurat_clusters"
NormalizeMetacells	
SetDatExpr	group.by = "seurat_clusters", assay = "RNA", slot = "data"
TestSoftPowers	networkType = "signed"
ConstructNetwork	soft_power = 7 for epithelium/ soft_power = 5 for HAND2+ PA, setDatExpr = FALSE
ScaleData	
ModuleEigengenes	group.by.vars = "seurat_clusters"
ModuleConnectivity	group.by = "seurat_clusters", sparse = FALSE

5) Gene Ontology enrichment analysis using clusterProfiler and org.Hs.eg.db

Function	Parameters
enrichGO	OrgDb = org.Hs.eg.db, keyType = "SYMBOL", ont = "BP" or "MF", pAdjustMethod = "bonferroni", pValueCutoff = 0.05, qValueCutoff = 0.05
simplify	cutoff = 0.7, by = "p.adjust", select_fun = min, measure = "Wang", semData = NULL

Supplementary Figures

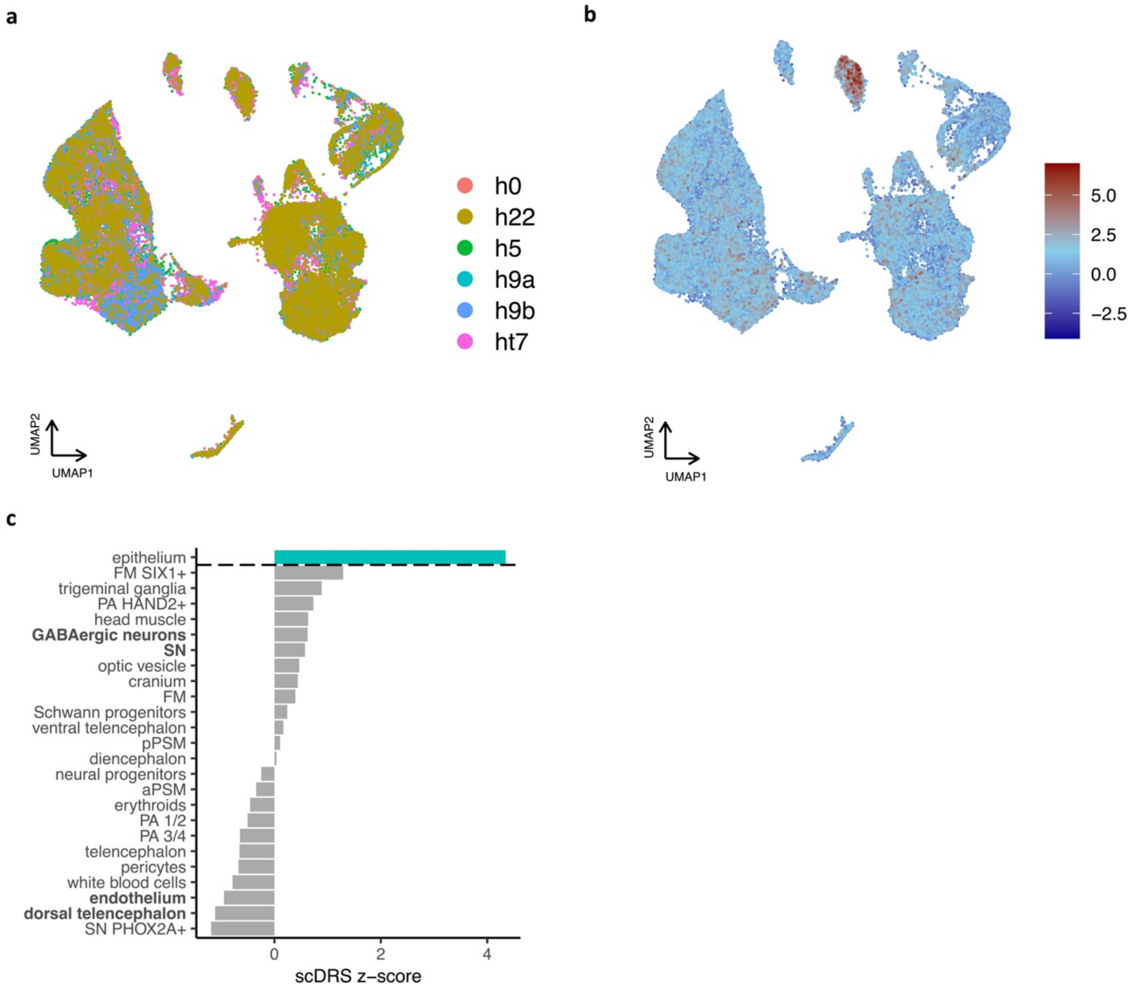

Figure S1. (a) UMAP plot from Fig. 1A colored according to sample ID of the embryos. **(b)** UMAP plot from Fig. 1A colored according to the scDRS disease association with nsCL/P at the single-cell level based on the weighted gene list. **(c)** scDRS disease association at the cell type level based on the weighted setting. Cell types above the dashed line showed significant association with the nsCL/P gene set. Bold cell type labels indicate significant within-cell type heterogeneity with respect to disease association. Anterior presomitic mesoderm (aPSM), Carnegie stage (CS) frontonasal mesenchyme (FM), $\log_2$ fold change ($\log_2$FC), non-syndromic cleft lip with/without cleft palate (nsCL/P), pharyngeal arches (PA), posterior presomitic mesoderm (pPSM), sympathetic neurons (SN), single-cell disease relevance score (scDRS).

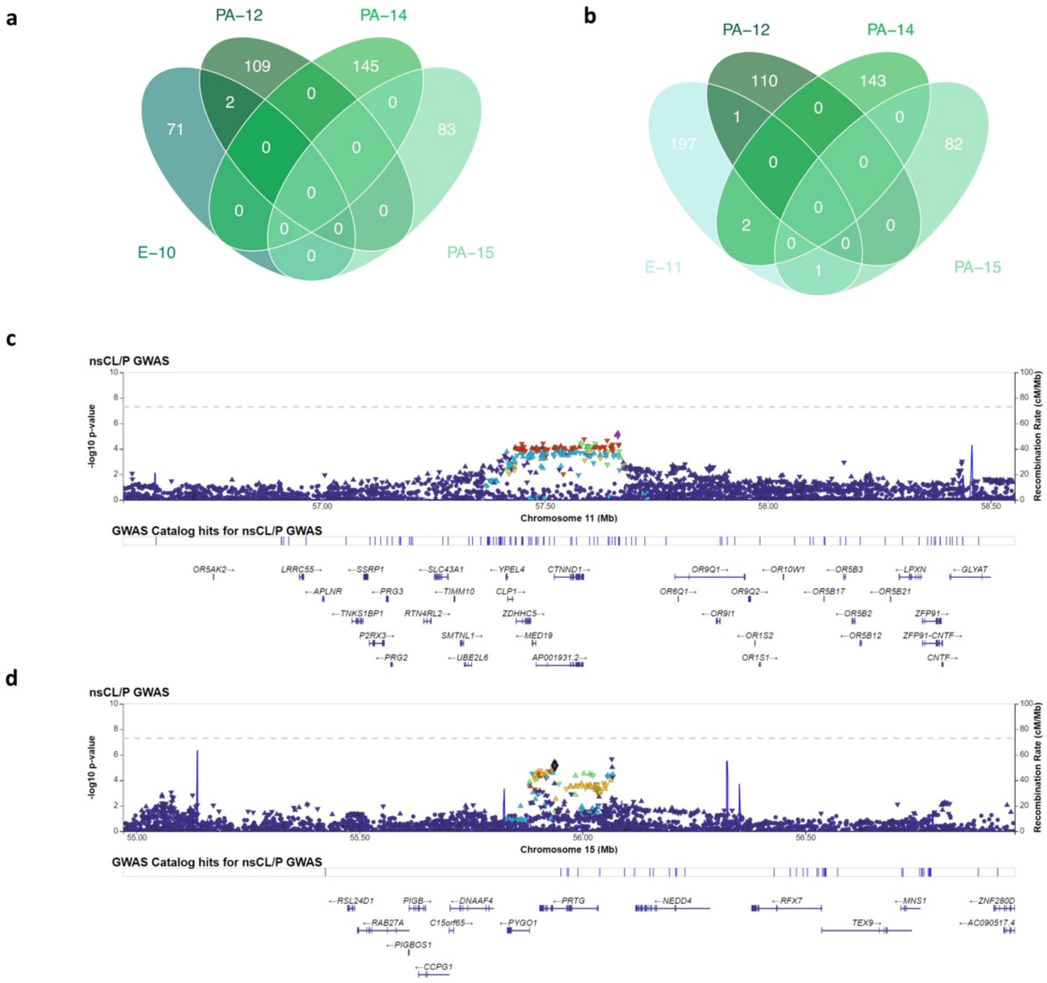

Figure S2. **(a)** Venn diagram of gene overlap between epithelial gene module E-10 and *HAND2*+ PA gene modules PA-12, PA-14, and PA-15. **(b)** Venn diagram of gene overlap between epithelial gene module E-11 and *HAND2*+ PA gene modules PA-12, PA-14, and PA-15 **(c)** LocusZoom plot of *CTNND1* from summary statistics of Welzenbach et al., 2021. **(d)** LocusZoom plot of *PRTG* from summary statistics of Welzenbach et al., 2021. Epithelium (E), genome-wide association study (GWAS), non-syndromic cleft lip with/without cleft palate (nsCL/P), pharyngeal arches (PA).

Supplementary Tables

All supplementary tables are provided in one Excel spread sheet. The abbreviations used in the supplementary tables are explained in the first sheet of the Excel spread sheet.

Table S1. Cluster marker genes of embryonic scRNA-seq data

Table S2. Differentially expressed genes between nsCL/P-associated and non-associated epithelial cells

Table S3. scDRS cell type level results for unweighted setting

Table S4. scDRS cell type level results for weighted setting

Table S5. scDRS gene level results for unweighted setting

Table S6. scDRS gene level results for weighted setting

Table S7. hdWGCNA co-expression gene modules for epithelium

Table S8. hdWGCNA co-expression gene modules for *HAND2*+ pharyngeal arches

Table S9. Hub genes of epithelial co-expression gene modules

Table S10. Gene ontology terms for epithelial and *HAND2*+ pharyngeal arches co-expression gene modules

Table S11. Hub genes of *HAND2*+ pharyngeal arches co-expression gene modules

Table S12. Overlap of epithelial and *HAND2*+ pharyngeal arches co-expression gene modules

Table S13. MAGMA gene set analysis results
